# Lecture on Clinical Medicine, Delivered at the Philadelphia Medical Institute

**Published:** 1838-08-01

**Authors:** W. W. Gerhard

**Affiliations:** Physician to the Philadelphia Hospital, &c.


### CLINICAL LECTURE.

LECTURE ON CLINICAL MEDICINE, *delivered at the Philadelphia Medical Institute, by* W. W. GERHARD, M. D., *Physician to the Philadelphia Hospital*, &c.

### DYSENTERY.

(Concluded from page 241.)

*Friday, July 13th.*—In my last lecture, I spoke to you of dysentery. I might have sketched different modes of treatment, as influencing the mortality in this disease, but I preferred waiting till you should have seen a number of cases. There are various general modes of treating it. All dysenteries vary in violence, and there is no simple set of symptoms, but the greatest variety; these correspond to the severity of the disorder, the rectum alone being affected, in many cases, while, in others, the whole mucous membrane of the alimentary canal is involved. This last is the ordinary form of the disease when it prevails as an epidemic, which is local, usually confined to particular districts. There was lately an epidemic at Germantown, which was very severe, though the city of Philadelphia has been healthy. Princeton, and its vicinity, were subject to epidemic attacks of dysentery of a very aggravated character, which yielded but little to medicine. The epidemic of last summer, which some of you witnessed, was the most severe that has occurred at Philadelphia for many years.

In France, very simple modes of treatment only are required; Broussais' plan of using opiates,—paralysing the bowels, putting them into splints, as it were, scarcely ever fails. In Paris, I never saw a fatal case of dysentery; and a gentleman there, who had charge of a large hospital, told me that, in many years, he had lost but three or four cases of it. In cases, where there are few nervous symptoms, and no ataxic condition of the system, (meaning by ataxic, severe prostration of strength,) we can readily cure by mere opiates, say twenty drops of laudanum, frequently repeated. They soothe the irritation; the narcotic checks the action of the bowels, and nature effects a cure. In severe cases, we must address our remedies to the skin, and make use of local depletion. Bleeding from the arm is rarely necessary; cataplasms should be applied over the bowels, and we may use leeches to the anus and abdomen. Depletion from the anus is very serviceable, as it acts almost immediately upon the inflamed surface.

There was a case shown to some of you, this morning, of a child, which had been attacked with measles, followed by dysentery; the disease was confined principally to the rectum and lower portion of the colon; the upper part of the colon was pale, and some false membrane spread over the rectum, and there was, also, some softening of the mucous membrane. Slight cases involve the rectum alone, the transverse or descending colon being never affected, and they are at once cured by leeches to the anus. In inflammatory cases, this sometimes acts like magic.

In some years, dysentery is more rapid in its course, and will not yield easily to remedies. I will now speak of the dysentery of warm climates. Warm weather, or, rather warm days, followed by cold nights, such as are common in tropical climates, produce the disease. The late change in the temperature has already produced several cases of dysentery; the thermometer having fallen twenty degrees, in the last two days. Chronic dysentery, for I have not seen the acute form in tropical climates, usually occurs from exposure to the night air, after very warm days. Sailors who have their watch on deck, during the night, are very subject to it.

Various kinds of treatment are serviceable in different stages of dysentery; as to which is the best, I am undecided. It is impossible to lay down any one plan for managing the disease, as we must modify our practice according to the particular character of the affection at the time. Last year it was very malignant, there was great disposition to gangrene, and fœtid sloughs were mingled with the stools. Post mortem examinations showed ulcerations and gangrene of the intestines. During life, there was great prostration and much subsultus. Depletion would not here have answered; in strong men, it might have been beneficial, but I am by no means sure of it. In typhoid cases, as they are called, (using the word typhoid in a very vague sense) we must attend particularly to the skin, and give mild stimuli; a prescription of Dr. Twining's I have found beneficial, consisting of

| | |
|---|---|
| Pulv. Ipecac. | gr. vi. |
| Ext. Gentian. | gr. iv. |
| Pilul. Hydrarg. | gr. v. |

Three times a day.

This was used towards the close of the disease and did much good; but little opium was given with it, and the discharges from the bowels became more feculent and less frequent. Our treatment did more good towards the close of the disease, in accordance with the rule that all epidemics become milder after a time. It is the case with fever and ague, yellow fever, cholera, &c. I could not decide as to the efficacy of this treatment in very severe cases. We used, besides this prescription, a combination of ipecacuanha, calomel, and opium, such as is employed in bronchitis, accompanied with great prostration. The calomel was increased from one-sixth to one-fourth, and one-half of a grain every two hours, when salivation was desired, and a grain of ipecacuanha, and from one to two grains of opium, according to the severity of the symptoms, were combined with it; in mild cases this treatment proved very serviceable. In the first prescription the ipecacuanha did the most good by its effect upon the skin; occasionally, but not frequently, vomiting was produced. Where the last prescription was used, it was evidently the mercury that acted, and the speedy relief of the symptoms, when the gums were touched, was very decided. In cases accompanied with much sloughing the mercury did not appear to be of great service.

There has been introduced in these cases, within a few years, a combination of sugar of lead and opium. The claim to the origination of this remedy is a disputed point; a very respectable physician, in extensive practice in this city,* first advocated it here. Dupuytren suggested its use in cholera, six months before the disease appeared in Paris, and treated a number of patients with it in a ward of one of the hospitals, and, after losing rather more than any one else, he gave it up.

Two years ago, this remedy, in my hands, answered better than last year in checking the hæmorrhage from the intestines, for the disease was not, at that time, attended with so much prostration as last year. The most severe case under my notice occurred in one of the house physicians; this gentleman took two grains of acetate of lead with a grain of opium every hour for several days, being, as you see, a very large quantity of each article, but it did little good; the opium was then increased to two grains every hour, for four hours, when he became drowsy and the discharges were less frequent, were not attended with so much pain, and diminished in quantity; he, finally, slept for some time. Very large doses of the sugar of lead may be given in severe cases, without producing any griping pain in the bowels, although it is otherwise in milder; and this does not appear to be owing to any carbonate of lead, that may be mingled with the salt, for, in all our good apothecary shops, it is kept in the form of crystals and powdered when wanted for use. Ipecacuanha or mercury is occasionally added to it.

Great benefit is derived from injections, in mild cases; but, when there is much irritation of the lower part of the bowels, they cannot be borne; even laudanum, added to a very minute portion of mucilage and thrown up, will excite immediate spasm and be rejected; the mechanical irritation of the pipe of the syringe will also forbid its use, so that in such cases the opium must be taken by the mouth, though it is not so beneficial as when it can be given by injection. Towards the close of the disease, injections of laudanum will be very serviceable. We may also apply narcotics to the skin either to the anus or over the abdomen; in children, a few drops of laudanum in a poultice, applied to the anus will act very readily. With adults not so much effect is produced, for the skin of children absorbs with much greater facility, than with grown persons. In adults a teaspoonful may be used, with but little effect, but, in an infant it is unsafe to begin with more than three drops poured on the poultice. We can increase the quantity, gradually, watching the effects of it.

There are also hygienic remedies deserving attention, food and medicine to support the strength. Brandy punch and toddy, port wine, or even claret are indispensable, when the disease is accompanied with much prostration. Brandy toddy, warm, may be given frequently, and in small doses, and is probably the best remedy in severe cases. Milk punch is applicable to mild forms of the disease, but, when there is much inflammation of the intestines, the digestion of the milk will prove injurious, as it coagulates in the stomach and by passing into the bowels, will increase the evil already existing. In chronic cases, port wine is serviceable, but it is too stimulating in the active stages of the disorder. In using these remedies you must be guided, in a great measure, by the feeling of the patient, and if he complain, after their use, of a burning heat and pain in the stomach, they must be discontinued, and mucilages resorted to; cold water alone, or with some mucilage in it, is very good; with regard to the choice of a mucilage you may be guided by the taste of the patient. The bene plant, slippery elm, rice, or gum arabic are all used, although differing somewhat in their effects, some being astringent and others slightly laxative; the taste of the patient is always the best criterion; towards the decline of the disease, broth must be given and biscuit soaked in wine and brandy, the diet must be principally farinaceous, as articles of this sort are more easily absorbed in the active stages of the disease. In chronic cases, this insipid food frequently disgusts, and it will be necessary to change it.

Last summer a number of experiments were performed, of which I hope to give an account in the course of this summer, with a view of testing the nature of the secretions, and they were all found to be alkaline; the saliva, the urine, the perspiration, and the evacuations from the bowels. I then gave, with a view to correct this state of things, sulphuric acid, of course very much diluted, and I have also used it this year; the saliva, urine, and perspiration became acid, but the discharges from the bowels continued of the same character and as frequent as before; there was less pain, but this was probably owing to the opium. I then abandoned the acid, and used opium alone.

During the last summer the mineral acids were very useful in New England, where there is a domestic prescription highly spoken of, in epidemic dysentery, consisting of vinegar and salt, which, no doubt, owes its efficacy to the acid; whether acids would effect a cure I have not determined, but I know that they alter the secretions very materially. Here, for some time, a mixture of Hope's has been found very beneficial, consisting of camphor and nitric acid, not enough to irritate, combined with laudanum, in sufficient amount to check the frequency of the discharges. In the quantity of opium to be administered, we must be guided by the effect it produces. When the tongue becomes dry, attended with stupor and prostration, it must be reduced, not when the prostration is the effect of the disease, but when the face becomes livid and turgid, opium is prejudicial, except in small doses. As palliatives, several means are to be used; opium is always serviceable to tranquillize the system and allay pain, when alone or in combination. A mixture of camphor and opium is frequently used by persons afflicted with this disease without consulting a physician; at night more especially is this required, in order to procure sleep; during the day, it is frequently better not to use it.

* Dr. Harlan.

Of the purgative mode of treatment, I am not disposed to think favourably, in severe cases; in slight cases, it is unquestionably very beneficial, but my own experience is against it as a general mode of treatment. Castor oil with laudanum is one of the best towards the close of the affection, it removes the scyballa from the colon, and, as is generally the case, slight inflammation, especially of the mucous membrane, is best removed by a slight stimulus. Mercurials I prefer in the treatment of dysentery, to what is called the saline treatment, always varying the remedies, according to the particular variety of the disease at the time. I know that the saline treatment has had some strong advocates, but I am not at all satisfied with it, not because I have not tried it, for I have tried every thing, but the results have not been such as to induce me to give it any preference. Finding dysentery to be so complicated a disease, and so protean in its aspect, and that it may be treated in so many different ways, you might be led to suppose that one mode of treatment was as good as another, and that the patient would do as well, if let alone. Such, however, is not the case; any plan of treatment, unless manifestly absurd, is better than none, and a very good guide to us will be the instincts of the patient; if he desires hot drinks, we may gratify him, or should he give a preference to iced water, it will not be found injurious. Opium may be given, either to excite narcotism, in a slight degree, or merely as a palliative.

The chronic form of dysentery is very common among persons who have had this disease, with great severity, in the tropics; it is accompanied with ulcerations in the colon, and the intermediate mucous membrane is of a dark slate colour. Chronic diarrhœa we may often suspect to proceed from the presence of tubercles; in France all chronic diarrhœa is regarded as arising from tubercles, but, in this country, though very frequently, it is not invariably the case. The mucous membrane is pale and softened, and the character of the fluids is changed. This alteration in the mucous membrane is a secondary and not a primary lesion. In such cases as these, mercury may be pushed to gentle salivation, and much benefit will be derived from sulphur-baths. A sea voyage is also very serviceable. Patients here, labouring under this disorder, frequently are sent to the Virginia springs, but the sulphur bath, made artificially, I am disposed to think more beneficial from our making it stronger. Cold or salt baths, according to the feelings of the patient, may also be used, with advantage. In chronic forms of the disease, we should abstain in a great degree, from internal remedies; the insipid articles of diet should be used, unless the disease remains stationary, in which case we may change it completely, substituting mutton chops, chicken, and wine, such as claret. A friend of mine, in Paris, who had laboured under the disease for a long time, and persevered in a farinaceous diet, and the use of opium, was cured by abandoning it and eating beef steaks, and drinking the old French wines. Port is a good wine, but here we seldom can procure it pure; the French wines are slightly acid and astringent, and answer a very good purpose. I have not spoken to you of diarrhœa, as a separate disorder, as it will come in in an after-part of the course, and is a subject involving extensive connections. It may occur either as a primary or as a secondary disease, in which case it is often attended with organic changes.

